# Evaluation of photodynamic therapy in adhesion protein expression

**DOI:** 10.3892/ol.2014.2149

**Published:** 2014-05-16

**Authors:** CRISTINA PACHECO-SOARES, MAIRA MAFTOU-COSTA, CAROLINA GENÚNCIO DA CUNHA MENEZES COSTA, ANDREZA CRISTINA DE SIQUEIRA SILVA, KAREN C.M. MORAES

**Affiliations:** 1 Laboratory of Dynamics of Cellular Compartments, University of Vale do Paraiba, Institute for Research and Development, São José dos Campos-SP 12244-000, Brazil; 2 Department of Pharmacology, Federal University of São Paulo, São Paulo-SP 04021-001, Brazil; 3 University of São Paulo, Institute of Biosciences of Rio Claro, Rio Claro-SP 13506-900, Brazil

**Keywords:** phthalocyanines, focal adhesion, β1-integrin, reverse transcription-polymerase chain reaction, cell culture

## Abstract

Photodynamic therapy (PDT) is a treatment modality that has clinical applications in both non-neoplastic and neoplastic diseases. PDT involves a light-sensitive compound (photosensitizer), light and molecular oxygen. This procedure may lead to several different cellular responses, including cell death. Alterations in the attachment of cancer cells to the substratum and to each other are important consequences of photodynamic treatment. PDT may lead to changes in the expression of cellular adhesion structure and cytoskeleton integrity, which are key factors in decreasing tumor metastatic potential. HEp-2 cells were photosensitized with aluminum phthalocyanine tetrasulfonate and zinc phthalocyanine, and the proteins β1-integrin and focal adhesion kinase (FAK) were assayed using fluorescence microscopy. The verification of expression changes in the genes for FAK and β1 integrin were performed by reverse transcription-polymerase chain reaction (RT-PCR). The results revealed that HEp-2 cells do not express β-integrin or FAK 12 h following PDT. It was concluded that the PDT reduces the adhesive ability of HEp-2 cells, inhibiting their metastatic potential. The present study aimed to analyze the changes in the expression and organization of cellular adhesion elements and the subsequent metastatic potential of HEp-2 cells following PDT treatment.

## Introduction

Photodynamic therapy (PDT) is a treatment modality for various tumors and non-malignant diseases, in which visible light is used to activate a photosensitizer (1,2). The precise mechanism of PDT on cells and tissues has not been fully elucidated. However, singlet oxygen generated following exposing the sensitizer to an appropriate light wavelength has been identified as the possible cytotoxic agent responsible for direct tumor cell damage or cell death (3).

Phthalocyanines belong to a second generation photosensitizer and are reported as the most effective drugs for PDT (4). Phthalocyanines constitute a large class of compounds with high extinction coefficients in the red spectral region (630 and 800 nm), which have been identified to present excellent tumor-localizing properties and high photosensitizing efficiency (5).

Cellular components are adhered to the extracellular matrix (ECM) and among them are cell adhesion proteins, which allow cell anchorage, survival, proliferation and migration. There are four main cell adhesion protein superfamilies, including integrins, selectins, immunoglobulins and cadherins (6). Integrins are ubiquitous glycoproteins that modulate cell adhesion to the ECM components, including collagen, fibronectin, laminin and vitronectin. These elements form a link between the extracellular environment and the cytoskeleton, through interactions with adaptor proteins that constitute focal adhesion contacts. In particular, integrins participate in the regulation of survival, proliferation, migration and differentiation (6).

It has been established that PDT produces changes in the ECM and to cell adhesion, which are largely dependent on the type of photosensitizer and the treatment doses (7); however, the mechanisms underlying this effect remain elusive. In one study, the cells subjected to PDT, using an hematoporphyrin derivative as a photosensitizing agent, required a longer time to adhere to a plastic substrate and a confluent layer of untreated cells when compared with the control group, suggesting that the damaging effects involve cytoskeletal proteins (8). Furthermore, cytoskeletal reorganization damage following photodynamic treatment has been reported in several other studies (1,9,10), and it has been observed that changes in the capacity of PDT-induced cell adhesion is accompanied by remodeling of actin filaments (11,12).

The present study aimed to investigate the adhesion process of the cell line HEp-2 (human laryngeal carcinoma) that have been subjected to PDT with the photosensitizing aluminum phthalocyanine tetrasulfonated (AlPcS_4_) and zinc phthalocyanine (ZnPc).

## Materials and methods

### Cell line

The HEp-2 human laryngeal cancer cells, (Adolfo Lutz Institute, São Paulo, Brazil) were cultured as a monolayer of cells in Dulbecco’s modified Eagle’s medium (DMEM), supplemented with 10% fetal bovine serum (FBS), penicillin (100 U/ml) and streptomycin (100 mM/ml; Gibco-BRL, Carlsbad, CA, USA).

### Chemicals

ZnPc, violet crystal, human collagen type IV, phalloidin-TRITC, anti-focal adhesion kinase (FAK) and anti-β1-integrin monoclonal antibodies were obtained from Sigma Chemical Co. (St. Louis, MO, USA). AlPcS_4_ was obtained from Frontier Scientific, Inc., (Logan, UT, USA). Mouse anti-rabbit fluorescein isothiocyanate (FITC)-conjugated and calcein-AM IgG, as well as primers for β1-integrin, FAK and β-actin, were obtained from Invitrogen Life Technologies (Carlsbad, CA, USA).

### Photodynamic therapy

The cells were exposed to the photosensitizers AlPcS4 (10 μM ml^−1^) or ZnPc (10 μM ml^−1^) for 1 h and were irradiated with an As-Ga-Al diode laser (wavelength, 650 nm; energy density, 4,5 J/cm^2^; Bio Wave LLLT Dual-Kondortech, São Carlos-SP, Brazil).

### Immunostaining

Tissue culture plates were coated overnight with human collagen type IV (5 μg/well) at room temperature under sterile conditions. The wells were washed with phosphate-buffered saline (PBS; Sigmal Chemical Co.) and non-specific binding sites were blocked with 100 μl of 0.2% bovine serum albumin (BSA; Sigma Chemical Co.) in DMEM (Gibco-BRL) for 90 min at 37°C. The wells were seeded with 500 μl of the appropriate cell suspension, 10^5^ cells/ml, and incubated at 37°C in a humidified 5% CO_2_ incubator for 24 h. The attached cells following incubation with AlPcS4 or ZnPc were irradiated and immediately the groups were separated at the times of 0 and 12 h, and cells were incubated in 4% paraformaldehyde (Sigma Chemical Co.) in PBS for 15 min at room temperature. Cells were permeabilized with 0.2% of Triton X-100 (Sigma Chemical Co.) and 4% paraformaldeyde in PBS for 10 min, and then blocked with 1% BSA solution in PBS for 30 min. Subsequently, cells were incubated with phalloidin-TRITC (1:100/1 h; Sigma Chemical Co.), mouse anti-human monoclonal antibody against β1-integrin (1:500/1 h) or mouse anti-human monoclonal antibody against FAK (1:500/1 h) (both Sigma Chemical Co.), and then incubated with the rabbit anti-mouse polyclonal secondary antibody conjugated with fluorescein (FITC; 1:1,000/1 h).

### Reverse transcription-polymerase chain reaction (RT-PCR)

Total cellular RNA was extracted by TRIzol (Invitrogen Life Technologies). Reverse transcription of 1 μg RNA was conducted using Taq Man^®^ reverse transcription reagents (Invitrogen Life Technologies) according to the manufacturer’s instructions. Equal amounts of cDNA (1/20 of the reaction volume) were subjected to PCR amplification using the following primers: β1-integrin: forward, 5′-GGACAGTGTGTTTGTAGGAAGAGG-3′ and reverse, 5′-GCACTGAACAGATTCTTTATGCTC-3′; FAK: forward, 5′-TGCAAGTAAGGAAATACAGTTTGG-3′ and reverse, 5′-CCACATACACACACCAAACATCATCCA-3′, and were then visualized by ethidium bromide-stained agarose gel (Sigma Chemical Co.) electrophoresis. RT-PCR was performed using standard conditions.

### Cell-cell adhesion assay

Following PDT, cells were incubated with calcein-AM (2 μM/30 min) and seeded (10^5^ cells/well) over a HEp-2 culture in a confluent monolayer in 96-well plates. Cells were incubated at 37°C for 2 h. Following this time, the non-adherent calcein-labeled cells were removed for washing with the culture medium. The attached cells were incubated for 12 and 24 h. The number of cells attached was determined at the end of each period using a Leica DMLB fluorescence microscope (Leica Microsystems, Milton Keynes, Buckinghamshire, UK).

### Cell-matrix adhesion assay

Cells submitted to treatment following the incubation periods, were seeded at a concentration of 10^5^ cells/well in coverslips coated with human collagen type IV and incubated at 37°C with DMEM with 2% FBS to allow adhesion. Following this period, the non-adherent cells were removed with PBS and fixed with 96% ethanol for 10 min at room temperature. The cells were incubated with 0.1% crystal violet (Sigma Chemical Co.) for 30 min. Excess dye was removed with distilled water and then 300 μl dimethyl sulfoxide (Sigma Chemical Co.) was added for extraction of the label. The optical density of the plates was read at 570 nm on a microplate reader (Packard SpectraCount; Packard BioScience Co., Meriden, CT, USA). Each experiment was run in triplicate.

### Statistical analyses

The data are presented as the mean ± standard deviation. All results presented a Gaussian distribution allowing the use of analysis of variance to compare means among the groups. P<0.05 was considered to indicate a statistically significant difference. To conduct the statistical analysis and graphics, GraphPad InStat^®^ and Microcal Origin^®^ 6.0 software were used, respectively.

## Results

### Effect of PDT on cell morphology

**Figure 1:**
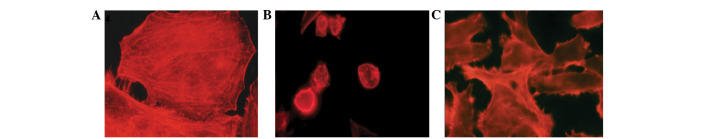
HEp-2 cells labeled with phalloidin-TRITC, 12 h following PDT. (A) The control group, non-irradiated cells with intact cytoskeleton (arrow); (B) the AlPcS_4_/PDT group, the filaments are concentrated in the cell periphery (arrow), with retraction of the cytoplasm; (C) in the ZnPc/PDT group, the disappearance of stress fibers of actin filaments (arrow) was observed. PDT, photodynamic therapy; AlPcS_4_, aluminum phthalocyanine tetrasulfonated; ZnPc, zinc phthalocyanine.

**Figure 2:**
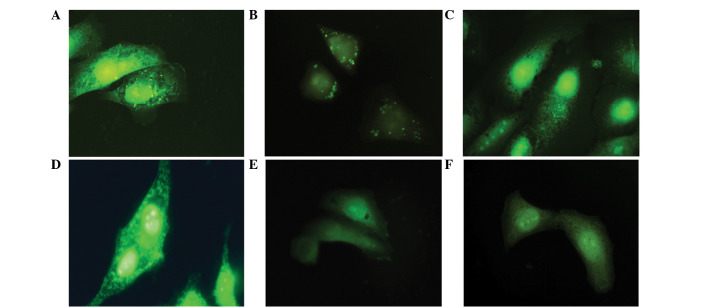
Immunostaining demonstrating β1-integrin and FAK expression in HEp-2 cells 12 h following PDT. The expression of β1-integrin in (A) the control group, cells with labeled in border (arrow); (B) the AlPcS_4_/PDT group, cells with diffuse labeling in the cytoplasm (arrow); (C) the ZnPc/PDT group, cells with some diffuse labeling in cytoplasm (arrow). FAC expression in (D) the control group, with numerous focal adhesion points (arrow); (E and F) the AlPcS_4_/PDT and ZnPc/PDT groups, where focal adhesion was not observed. PDT, photodynamic therapy; AlPcS_4_, aluminum phthalocyanine tetrasulfonated; ZnPc, zinc phthalocyanine; FAK, focal adhesion kinase.

Immunostaining analysis revealed that PDT acted on the actin filaments of the cytoskeleton and the adhesion proteins β1-integrin and FAK. A total of 12 h following PDT, immunostaining analysis observations of the control group revealed a homogeneous distribution of actin filaments with stress fiber characteristics (Fig. 1A). Following PDT, intense retraction in the actin filaments was observed in the AlPcS_4_ and ZnPc groups when compared with control group, revealing severe damage that was compromising the cellular morphology and the loss of integrity of the filaments with the disappearance of stress fibers (Fig. 1B and C). This consequently led to damage to the internal organization, mechanical and structural stability of the cells. Cell adhesion features and changes in commitment, adhesive proteins β1-integrin and FAK are illustrated in Fig. 2. Immediately following treatment, the cells were labeled for these adhesive proteins, but after 12 h occur a reduction of the same, when compared with the control group.

### Effect of PDT on adhesion protein expression

**Figure 3:**
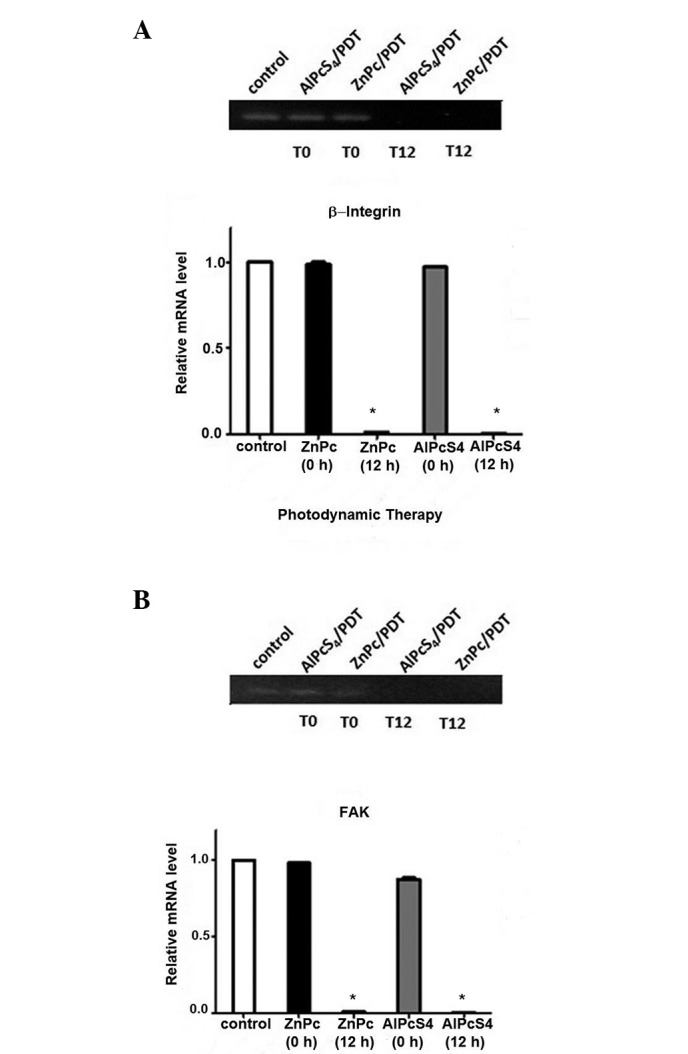
Effect of PDT on the mRNA expression of adhesion proteins. (A) There was no significant reduction in the mRNA expression of β1-integrin in the two groups when compared with the control group at 0 h. Following 12 h from PDT, there was a significant reduction in the expression in the ZnPc and AlPcS_4_ groups, when compared with the control group. (B) There was no significant difference in the expression of FAK mRNA for the two groups when compared with the control group at 0 h. By contrast, 12 h following PDT, a significant reduction in the expression of FAK was observed in the ZnPc and AlPcS_4_ groups when compared with the control group. PDT, photodynamic therapy; AlPcS_4_, aluminum phthalocyanine tetrasulfonated; ZnPc, zinc phthalocyanine; FAK, focal adhesion kinase.

The results observed in the immunostaining were confirmed by the analysis of the protein expression of FAK and β1-integrin following PDT. The expression of the adhesion proteins FAK and β1-integrin following PDT was assessed by RT-PCR. The analysis of the β1-integrin mRNA expression in the AlPcS_4_ and ZnPc groups compared with the control group revealed no significant differences at the baseline time (0 h; Fig. 3). However, 12 h following PDT, a significant reduction in the expression of β1-integrin in the ZnPc and AlPcS_4_ groups, as compared with the controls, was observed. The expression of FAK mRNA in the two groups did not demonstrate a significant reduction when compared with the control group at 0 h. By contrast, 12 h following PDT there was a significant reduction in the FAK mRNA expression in the ZnPc and AlPcS_4_ groups, when compared with the control group. Therefore, the two photosensitizers used demonstrated efficacy in reducing the expression of β1-integrin and FAK, influencing the accession process following PDT.

### Effect of PDT on cell-cell adhesion

**Figure 4:**
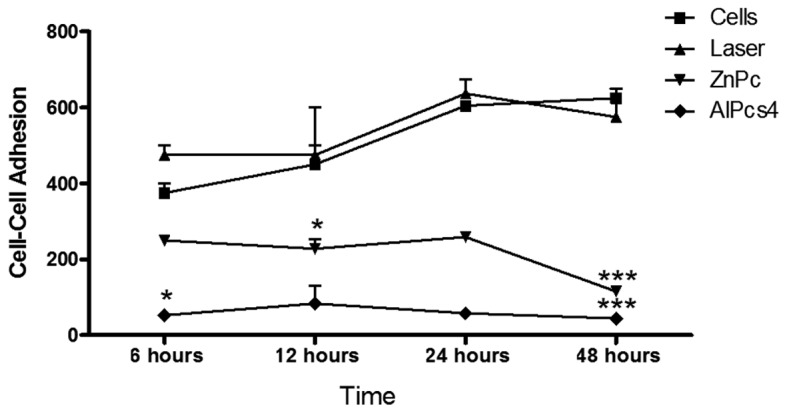
Cell-cell adhesion assay. Following PDT, the cells were incubated for periods of 6, 12, 24 and 48 h. At the end of these periods the cultures were analyzed by fluorescence microscopy, counting the number of cells adhered to the monolayer. After 24 h, the difference in the adhesion ability in the treatment groups, compared with the control, was significant (^***^P<0.001). When comparing the photosensitizers ZnPc and AlPcS_4_, there was no significant difference. PDT, photodynamic therapy; AlPcS_4_, aluminum phthalocyanine tetrasulfonated; ZnPc, zinc phthalocyanine.

To study cell-cell interactions, calcein-AM was used as an indicator of cell viability. The control and treatment groups were incubated with calcein-AM and cocultured in HEp-2 monolayer cells. The cells were incubated for 6, 12, 24 and 48 h to evaluate the adhesion ability following treatment with PDT. The behavior of the laser and control groups at all times demonstrated an increase in cell adhesion throughout the period analyzed. Cultures submitted to photodynamic treatment after 6, 12, 24 and 48 h (Fig. 4) exhibited a marked reduction in the number of cells adhered to monolayer compared with the cells in the control group. In the ZnPc PDT group, the adherence rates were low at 6 h, but demonstrated no significant change until 24 h, with a significant reduction (P<0.001) observed at 48 h. In the PDT-AlPcS_4_ group, <100 cells were attached in the same period.

### Effect of PDT on cell adhesion to the matrix

**Figure 5:**
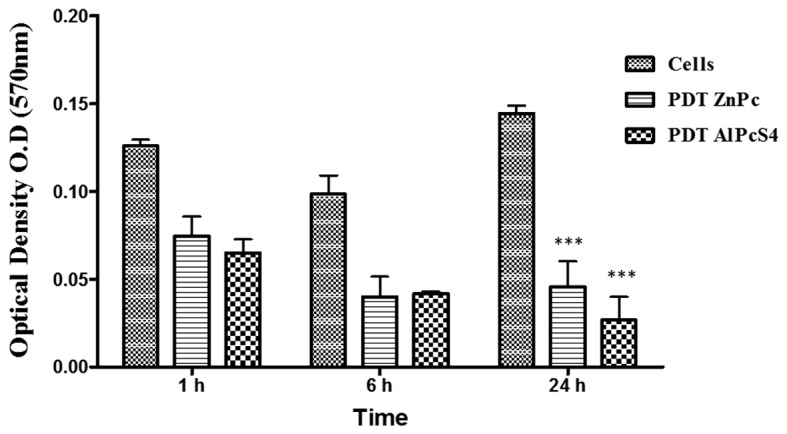
Cell-matrix adhesion assay. Following PDT, the cultures were incubated in collagen matrix IV at 1, 6 and 24 h, and analyzed by a colorimetric assay with crystal violet. In the ZnPc/PDT group, the adherence rates initially (6 h) were low, but not significantly different from the control group (^*^P>0.05). Following 12 h, a significant reduction was observed (P<0.05) and from 24 h the reduction was significant compared with the control group (^***^P<0.001). PDT, photodynamic therapy; AlPcS_4_, aluminum phthalocyanine tetrasulfonated; ZnPc, zinc phthalocyanine.

The capacity of cells to adhere to matrix, was evaluated using a colorimetric assay. Comparing the ZnPc/PDT and AlPcS_4_/PDT groups with the controls, there was a significant reduction in the number of cells adhered (P<0.01) at the 1 and 6 h time points following incubation of the cells with the matrix. After 24 h, this difference was significant (P<0.001). There was no significant difference in cell adhesion when comparing the two photosensitizers ZnPc and AlPcS_4_ (Fig. 5).

## Discussion

The present study describes the effect of photosensitizers ALPcS_4_ and ZnPc on cell adhesion. AlPcS4 was found to modify the structure of actin filaments, with more severe changes identified during the periods of 12, 24 and 48 h, as demonstrated by labeling with phalloidin-TRITC. In cultures treated with ZnPc/PDT, it was possible to observe the presence of small cytoplasmic projections, with concentrated actin filaments in the cell edge, demonstrating that cellular organization was affected by PDT treatment following 12 h, with a marginal recovery at 48 h. The cellular structure of actin is recognized as crucial to the maintenance of cell adhesion, and is therefore one of the targets of PDT (13,14); however, changes induced by PDT in the cytoskeletal proteins may be present in cells resistant to treatment, leading to changes in the adhesion and organization of the cytoskeletal components favoring the migration of these cells to other tissues. This alteration may be explained by the involvement of adhesion proteins, mainly β1-integrin and FAK, as these proteins are dependent on the disposition of the actin filaments. In the present study, the immunostaining of β1-integrin and FAK demonstrated a reduction in their protein expression 12 h following PDT. The RT-PCR results confirmed the reduction of the mRNA expression β1-integrin and FAK, 12 h following PDT for the two photosensitizers. These results were corroborated in the study by Milla Sanabria *et al* (6), which suggested that the adhesion of the cell to the substratum is mediated by integrins, and would therefore be interrupted following photodynamic action by damage to the ECM and by damage directly to the integrin proteins.

The stability of integrin (α and β) and FAK is dependent on the interaction with other proteins, such as vinculin, paxillin and actin filaments. In the present study, it was verified that the mRNA expression for adhesion proteins was reduced following PDT. This result indicates that PDT is not only acting to destabilize the interaction between the proteins involved in cell-substrate adhesion, as observed in the results of the cell-matrix interaction, but that it is also acting on signaling pathways in cells. The data of the violet crystal assay demonstrated a significant reduction in cell-matrix adhesion 24 h following PDT for the two phthalocyanines. This effect was also observed in cell-cell interactions in which a reduction of adhesion was observed following PDT, which was enhanced as the time increased. These results confirm earlier evidence from Runnels *et al* 1999 (15), who performed a similar study assessing the adhesion to collagen IV, laminin, fibronectin and vitronectin (components of ECM) with PDT-AlPcS4. The reduction of cell adhesion OVCAR 3 (human ovarian carcinoma) subjected to treatment with BPD-MA was attributed to the high rate of cell death observed in culture; however, even with decreasing rates of β1-integrin in the focal adhesion plaques, there were no differences in the expression of this protein.

In conclusion, the phthalocyanines AlPcS_4_ and ZnPc, following irradiation, induce damage that compromises the cell adhesion ability and inhibits the metastatic potential of HEp-2 cells. However, further studies are required to determine the signaling pathways involved in the resistance of the surviving cells.

## References

[1] Juarranz A, Espada J, Stockert JC (2001). Photodamage induced by Zinc(II)- phthalocyanine to microtubules, actin, alpha-actinin and keratin of HeLa cells. Photochem Photobiol.

[2] Dolmans DE, Fukumura D, Jain RK (2003). Photodynamic therapy for cancer. Nat Rev Cancer.

[3] Allison RR, Moghissi K (2013). Photodynamic Therapy (PDT): PDT Mechanisms. Clin Endosc.

[4] Dougherty TJ, Gomer CJ, Henderson BW (1998). Photodynamic therapy. J Natl Cancer Inst.

[5] Castano AP, Demidova TN, Hamblin MR (2005). Mechanisms in photodynamic therapy: Part three-Photosensitizer pharmacokinetics, biodistribution, tumor localization and modes of tumor destruction. Photodiagn Photodyn Ther.

[6] Milla Sanabria L, Rodríguez ME, Cogno IS (2013). Direct and indirect photodynamic therapy effects on the cellular and molecular components of the tumor microenvironment. Biochim Biophys Acta.

[7] Pazos MD, Nader HB (2007). Effect of photodynamic therapy on the extracellular matrix and associated components. Braz J Med Biol Res.

[8] Uzdensky A, Kolpakova E, Juzeniene A, Juzenas P, Moan J (2005). The effect of sublethal ALA-PDT on the cytoskeleton and adhesion of cultured human cancer cells. Biochim Biophys Acta.

[9] Ferreira SDRM, Tedesco AC, Sousa G (2004). Analysis of mitochondria, endoplasmic reticulum and actin filaments after PDT with AlPcS(4). Lasers Med Sci.

[10] Tsai T, Ji HT, Chiang PC, Chou RH (2009). ALA-PDT results in phenotypic changes and decreased cellular invasion in surviving cancer cells. Lasers Surg Med.

[11] Machado AH, Moraes KC, Pacheco-Soares C (2010). Cellular changes after photodynamic therapy on HEp-2 cells using the new ZnPcBr(8) phthalocyanine. Photomed Laser Surg.

[12] Milla LN, Cogno IS, Rodríguez ME, Sanz-Rodríguez F, Zammarrón A, Gilaberte Y, Carrasco E, Rivarola V, Juarranz A (2011). Isolation and characterization of squamous carcinoma cells resistant to photodynamic therapy. J Cell Biochem.

[13] Liu T, Wu LY, Berkman CE (2010). Prostate-specific membrane antigen-targeted photodynamic therapy induces rapid cytoskeletal disruption. Cancer Lett.

[14] Casas A, Sanz-Rodriguez F, Di Venosa G (2008). Disorganisation of cytoskeleton in cells resistant to photodynamic treatment with decreased metastatic phenotype. Cancer Lett.

[15] Runnels JM, Chen N, Ortel B (1999). BPD-MA-mediated photosensitization in vitro and in vivo: cellular adhesion and beta1 integrin expression in ovarian cancer cells. Br J Cancer.

